# MicroRNA Signature in Plasma-Derived Extracellular Vesicles in Frontotemporal Dementia

**DOI:** 10.3390/genes17080945

**Published:** 2026-08-13

**Authors:** Evelyne Minucchi, Francesca Dragoni, Rosalinda Di Gerlando, Gaia Pavanello, Matteo Cotta Ramusino, Alfredo Costa, Stella Gagliardi

**Affiliations:** 1 Department of Brain and Behavioral Sciences, University of Pavia, 27100 Pavia, Italy; evelyne.minucchi@mondino.it (E.M.); alfredo.costa@mondino.it (A.C.); 2 Molecular Biology and Transcriptomics Unit, IRCCS Mondino Foundation, 27100 Pavia, Italy; rosalinda.digerlando01@universitadipavia.it (R.D.G.); stella.gagliardi@mondino.it (S.G.); 3 Department of Biology and Biotechnology “Lazzaro Spallanzani”, University of Pavia, 27100 Pavia, Italy; gaia.pavanello01@universitadipavia.it; 4 Unit of Behavioral Neurology and Dementia Research Center, IRCCS Mondino Foundation, 27100 Pavia, Italy; matteo.cottaramusino@mondino.it

**Keywords:** frontotemporal dementia, FTD, microRNA, extracellular vesicles, EV, biomarkers

## Abstract

**Background:** Frontotemporal dementia (FTD) is a neurodegenerative disease that shares numerous clinical features with other forms of dementia. In this context, non-coding RNAs, specifically microRNAs (miRNAs), represent a promising tool for differential diagnosis. Since these miRNAs can be isolated from circulating extracellular vesicles (EVs) in peripheral blood, they provide a direct insight into FTD-specific molecular processes. Consequently, while EV-contained miRNAs hold potential as disease-specific biomarkers, investigating their relative target genes can help elucidate their precise functional roles. **Aim**: This work aimed to identify a specific miRNA signature to better characterize FTD pathology. **Methods**: Building on a previous Next-Generation Sequencing (NGS) analysis, three candidate miRNAs were selected for validation in both EVs and peripheral blood mononuclear cells (PBMCs) of FTD patients. Subsequently, the predicted target genes of two of these miRNAs were validated in PBMCs to assess their expression levels. **Results**: Our findings revealed that miR-365a-3p and miR-212 were significantly down-regulated in FTD. **Conclusions**: Together with their target genes, these miRNAs are involved in cell cycle and apoptotic pathways, suggesting a potential role in the pathological mechanisms of the disease.

## 1. Introduction

Frontotemporal dementia (FTD) represents a heterogeneous class of neurodegenerative disorders characterized by the progressive degeneration of the frontal and temporal lobes, standing as a prominent cause of mortality among the elderly population [1,2]. Diagnosis is often challenging because the disease shares clinical features with several mood and psychiatric disorders, leading to frequent diagnostic overlap [3]. The clinical onset typically manifests around 56 years of age, with a life expectancy ranging between 7 and 13 years post-diagnosis [4,5]. While the majority of FTD cases occur sporadically, a subset of patients exhibits an autosomal dominant inheritance pattern [6]. At the histopathological level, FTD displays marked neuropathological diversity, classified according to the specific protein inclusions accumulated within neurons and glial cells [7]. To date, the three primary proteins implicated in the pathogenesis of the disease are Tau, TAR DNA-binding protein 43 (TDP-43), and Fused in Sarcoma (Fus) [8,9].

Given the extensive clinical overlap between FTD variants and other forms of dementia, identifying robust fluid-based biomarkers is paramount to discerning distinct molecular signatures characteristic of this specific neurodegenerative pathology. Consequently, recent research has increasingly focused on Extracellular Vesicles (EVs), and specifically on their molecular cargo, as promising biomarker candidates [10]. EVs are cell-derived, membrane-bound structures classified by size into small extracellular vesicles (SEVs, 30–130 nm) and large extracellular vesicles (LEVs, 130–1000 nm in diameter) [11]. Notably, EVs are capable of crossing the blood–brain barrier (BBB); hence, their isolation from peripheral blood offers a minimally invasive and reliable reflection of the central nervous system’s pathological state [12]. Among the diverse molecular components of the EV cargo, small non-coding RNAs have emerged as particularly compelling biomarkers [13]. Within this class, microRNAs (miRNAs) act as crucial post-transcriptional regulators of gene expression by binding to the 3′ untranslated region (3′ UTR) of target mRNAs, ultimately inducing translational repression or gene silencing [14]. Crucially, encapsulation within the lipid bilayer of EVs shields these miRNAs from extracellular RNase-mediated degradation, imparting superior stability compared to free circulating miRNAs and enhancing their reliability as diagnostic biomarkers [15]. Alterations in miRNA expression profiles can reflect the underlying pathological state of an individual, and their dysregulation is known to drive the progression of various disorders, including FTD [16]. A primary consequence of aberrant miRNA expression is the downstream dysregulation of their respective target genes, which intricately compromises relevant pathophysiological pathways [17]. Despite the escalating interest in EV-associated miRNAs as biomarkers for neurodegenerative diseases, their specific role in FTD remains largely uncharacterized. Indeed, while the extensive literature documents miRNA profiles in dementias such as Alzheimer’s disease (AD), data regarding these molecules in FTD remain scarce.

This project addresses the critical need to identify a specific miRNA signature for FTD, thereby broadening the understanding of the molecular mechanisms underlying the pathology through the investigation of their downstream target genes. Drawing upon data from a sequencing study on plasma-derived EVs aimed at characterizing SEV-derived miRNAs in neurodegenerative disorders [18], we selected and validated a panel of differentially expressed miRNAs in both EVs and peripheral blood mononuclear cells (PBMCs). The subsequent phase of the study involved the identification and bioinformatic analysis of the target genes regulated by these validated miRNAs, aimed at elucidating their potential involvement in the progression of FTD.

## 2. Materials and Methods

### 2.1. Cohort Selection

Blood samples were collected from 26 FTD patients and 26 age- and sex-matched (CTRL) individuals. The sample size was based on the availability of eligible samples in our laboratory at the time of the study. Therefore, no formal a priori sample size calculation was performed as this was an exploratory study.

A total of 26 FTD patients were enrolled from the Behavioral Neurology Unit of the IRCSS Mondino Foundation (Pavia, Italy), referred to our Institute between May 2020 and November 2024. We included subjects aged 50 to 80 years with diagnosis of a behavioral variant of frontotemporal dementia (bvFTD) [19] or motor neuron disease (MND) [20] or a non-fluent variant of Primary Progressive Aphasia (nfvPPA) [21]. Subjects with psychiatric disease, epilepsy or any uncontrolled medical condition that could contribute to cognitive or behavioral impairment (e.g., nephropathy, liver disease, brain tumor, alcohol or drug abuse, or normal pressure hydrocephalus) were excluded. Enrolled patients underwent complete clinical, neurological and neuropsychological assessment, and brain magnetic resonance imaging (MRI). The neuropsychological examination included tests for global cognitive efficiency (Mini Mental State Examination, MMSE), and for the following domains: memory, logical and executive functioning, attention, language and visual–spatial perception.

The 26 CTRL individuals were recruited at Transfusional Service and Centre of Transplantation Immunology (San Matteo Foundation IRCCS, Pavia, Italy). All subjects included in this study signed an informed consent (Protocol No. C.E. 4407/22). The study was conducted in accordance with the ethical principles outlined in the Declaration of Helsinki.

Details on the subjects included in the study are reported in Table 1 and Table S1.

### 2.2. EV Isolation and RNA Extraction

Blood was collected in sodium citrate tubes, and then plasma was isolated. EVs were isolated from plasma using Total Exosome Isolation Kit (ThermoFisher, Waltham, MA, USA) starting from the same volume of plasma for all samples (1 mL), ensuring comparable input material across experimental groups. EV characterization was performed through nanoparticle tracking analysis (NTA) and transmission electron microscopy (TEM) analyses as described in our previous work [18]. RNA was extracted from EVs using the miRNeasy Serum/Plasma kit (Qiagen, Hilden, Germany) according to the manufacturer’s instructions. The concentration and the quality of the samples were assessed using Nanodrop (ThermoFisher, Waltham, MA, USA).

### 2.3. PBMCs Isolation and RNA Extraction

Starting from peripheral venous blood samples, PBMCs were isolated using Histopaque^®^-1077 (Sigma-Aldrich, St. Louis, MO, USA). Total RNA was then extracted from 5 × 10^6^ PBMCs pellet using the miRNeasy Tissue/Cells Advanced Mini Kit (Qiagen, Hilden, Germany) according to the manufacturer’s instructions. The concentration and the quality of the samples were assessed using Nanodrop (ThermoFisher, Waltham, MA, USA).

### 2.4. Deregulated miRNA Selection and Two-Step Validation

In a previous transcriptomic analysis, Di Gerlando et al. (2026) [18] performed the processing of the obtained dataset using bcl2fastq2 Conversion Software (v2.20; Illumina, San Diego, CA, USA) and docker4seq workflow [18] and performed a differential expression analysis using the EBSeq package in R. MiRNAs were considered differentially expressed when they showed a false discovery rate (FDR) ≤ 0.1 and |log_2_FC| > 1. Statistical significance was assessed after Benjamini–Hochberg correction for multiple testing (adjusted *p*-value < 0.05).

In this cited dataset, Rosalinda et al. identified a total of 340 differentially expressed (DE) miRNAs in FTD patients. From this data collection, specific candidate miRNAs were selected for further investigation based on one of the two following criteria:

1. A comprehensive review of the dementia-related literature and a stringent expression threshold their log_2_FC (|log_2_FC| ≥ 6);
2. A well-documented strong interaction with miRNAs chosen based on the first selection criteria.

These selected candidates, along with their respective assay IDs, are detailed in Table 2. Notably, two miRNAs (miR-365a-3p and miR-212-3p) satisfied the first selection criteria, whereas miR-132 was specifically chosen due to its well-documented, strong interaction with miR-212-3p. To confirm the high-throughput sequencing findings, these candidate miRNAs underwent a technical validation phase in extracellular vesicles (EVs) via reverse transcription quantitative polymerase chain reaction (RT-qPCR). This was performed using the TaqMan MicroRNA Assay (ThermoFisher, Waltham, MA, USA) in strict accordance with the manufacturer’s instructions.

Following the technical verification, a functional validation step was carried out in peripheral blood mononuclear cells (PBMCs). This subsequent phase focused exclusively on the two miRNAs (miR-132 and miR-365a-3p) that exhibited highly coherent expression profiles between the initial next-generation sequencing (NGS) data and the technical validation results in EVs. The expression levels of these target miRNAs in PBMCs were similarly quantified by RT-qPCR using the TaqMan MicroRNA Assay (ThermoFisher, Waltham, MA, USA).

Reverse transcription was performed using the MultiScribe^TM^ Reverse Transcriptase (ThermoFisher, Waltham, MA, USA) with 70 ng of RNA in a final reaction volume of 15 µL. Reverse transcription conditions were 16 °C for 3 min, 42 °C for 30 min and 85 °C for 5 min.

Preamplification reaction was performed in a finale volume of 25 µL using TaqMan PreAmp Master Mix 2X (ThermoFisher, Waltham, MA, USA). Preamplification conditions were 95 °C for 10 min, 55 °C for 2 min, 72 °C for 2 min, 12 amplification cycles at 95 °C for 15 s, 60 °C for 4 min, and 99.9 °C for 1 min.

To evaluate expression levels, all selected target mRNAs were validated in EVs via RT-qPCR using the PCR Master Mix (ThermoFisher, Waltham, USA) in a final reaction volume of 20 µL in compliance with the manufacturer’s protocol. RT-qPCR reactions were performed in duplicate, and the cycling conditions were 95 °C for 20 s followed by 40 cycles of 95 °C for 3 s and 60 °C for 30 s. Fold-expression differences between CTRL and FTD groups were determined using the 2^−∆∆Ct^ method.

For quantitative normalization, U6 snRNA and miR-16-5p were employed as endogenous reference controls.

### 2.5. Target mRNA Selection and Validation

Downstream mRNA targets were selected for the two candidate miRNAs (miR-132 and miR-365a-3p) that had previously demonstrated the most robust and coherent expression profiles across both the EV and PBMC validation phases. Potential targets were initially screened utilizing the miRTarBase database [22].

1. From this cohort, the optimal mRNA targets were prioritized based on two stringent criteria.
2. Experimental Evidence: candidate interactions required strong, experimentally validated literature evidence of miRNA-mRNA binding within human cell lines.
3. Pathophysiological Relevance: Target genes had to be actively involved in biological pathways associated with neurodegeneration, verified through functional interaction networks using the STRING database [23].

Following this bioinformatic stratification, the final selection of target mRNAs comprised:

- *CCNB1* (Cyclin B1) and *FOXO3a* (Forkhead Box O3a) for miR-132;
- *CCND1* (Cyclin D1) and *SHC1* (SHC Adaptor Protein 1) for miR-365a-3p.

Reverse transcription was performed using the iScript™ cDNA Synthesis Kit (BioRad, Hercules, CA, USA) with 500 ng of total RNA in a final reaction volume of 20 µL. Reverse transcription conditions were 25 °C for 5 min, 42 °C for 30 min and 85 °C for 5 min.

To evaluate expression levels, all selected target mRNAs were validated in PBMCs via RT-qPCR using the iQ™ SYBR^®^ Green Supermix (BioRad, Hercules, USA) in a final reaction volume of 15 µL in compliance with the manufacturer’s protocol. RT-qPCR reactions were performed in duplicate, and the cycling conditions were 95 °C for 3 min followed by 40 cycles of 95 °C for 15 s and 63 °C for 45 s. Melting curve analysis was performed at the end of each amplification run to verify amplification specificity. All reactions showed a single melting peak, indicating the absence of non-specific amplification and primer–dimer formation. Fold-expression differences between CTRL and FTD groups were determined using the 2^−∆∆Ct^ method.

The comprehensive inventory of these validated targets, along with their respective oligonucleotide primer sequences, is detailed in Table S2. Quantitative normalization was performed using *GAPDH* as the endogenous reference gene.

### 2.6. Statistical Analysis

Statistical analyses were performed using the Mann–Whitney test and Spearman’s correlation using Prism GraphPad v9 software (GraphPad Software). Samples showing undetectable or unstable expression (i.e., inconsistent Ct values) were excluded from the analysis. For all analyses, *p* < 0.05 was considered statistically significant. Details on the statistical analysis with relative *p*-values are provided in Table S3A–C.

## 3. Results

### 3.1. miRNA Selection and Pathway Analysis

MiRNAs were chosen from a previous NGS analysis based on their log_2_FC (|log_2_FC| ≥ 6) [18] and the literature that had previously described them as deregulated miRNAs in neurodegenerative diseases. NGS analysis revealed that hsa-miR-365a-3p had a log_2_FC = −6036 and has-miR-212-3p had a log_2_FC = −6839, while has-miR-132 was chosen based on its wide descripted relationship with miR-212-3p.

**Figure 1:**
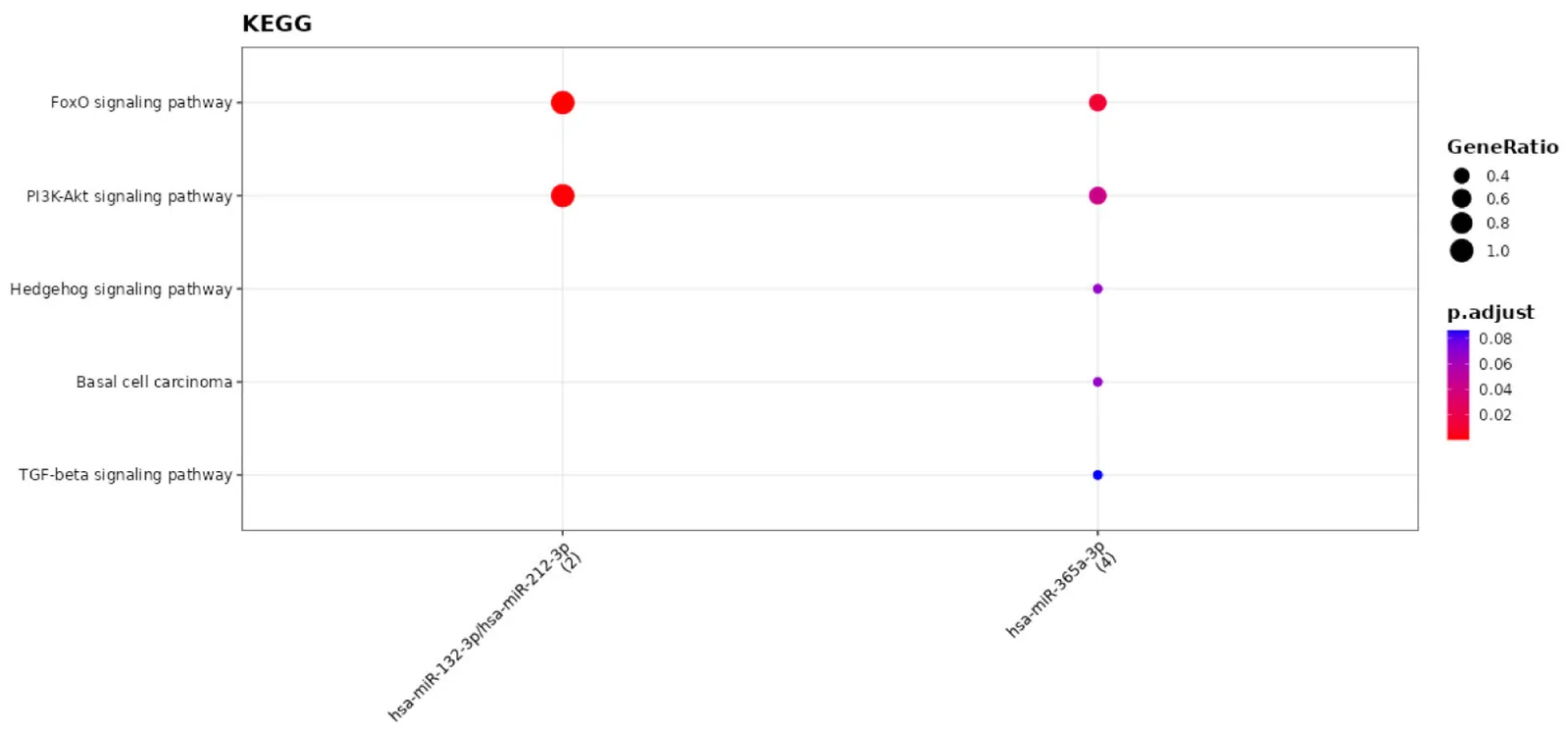
KEGG pathway analysis of miR-212-3p, miR-132 and miR-365a-3p obtained using Mienturnet [24].

A KEGG pathway analysis was conducted using Mienturnet tool (https://bio.tools/MIENTURNET, accessed on 7 August 2026) to clarify the biological process the selected miRNAs are involved with (Figure 1). The analysis revealed two pathways with significant *p*-value shared across the two miRNAs: the FoxO signaling pathway (*p*-value = 0.0002 for miR-212-3p and miR-132, *p*-value = 0.00118 for miR-365a-3p) and PI3KAkt signaling pathway (*p*-value = 0.00149 for miR-212-3p and miR-132, *p*-value = 0.00848 for miR-365a-3p).

### 3.2. DE miRNA Validation in SEVs

**Figure 2:**
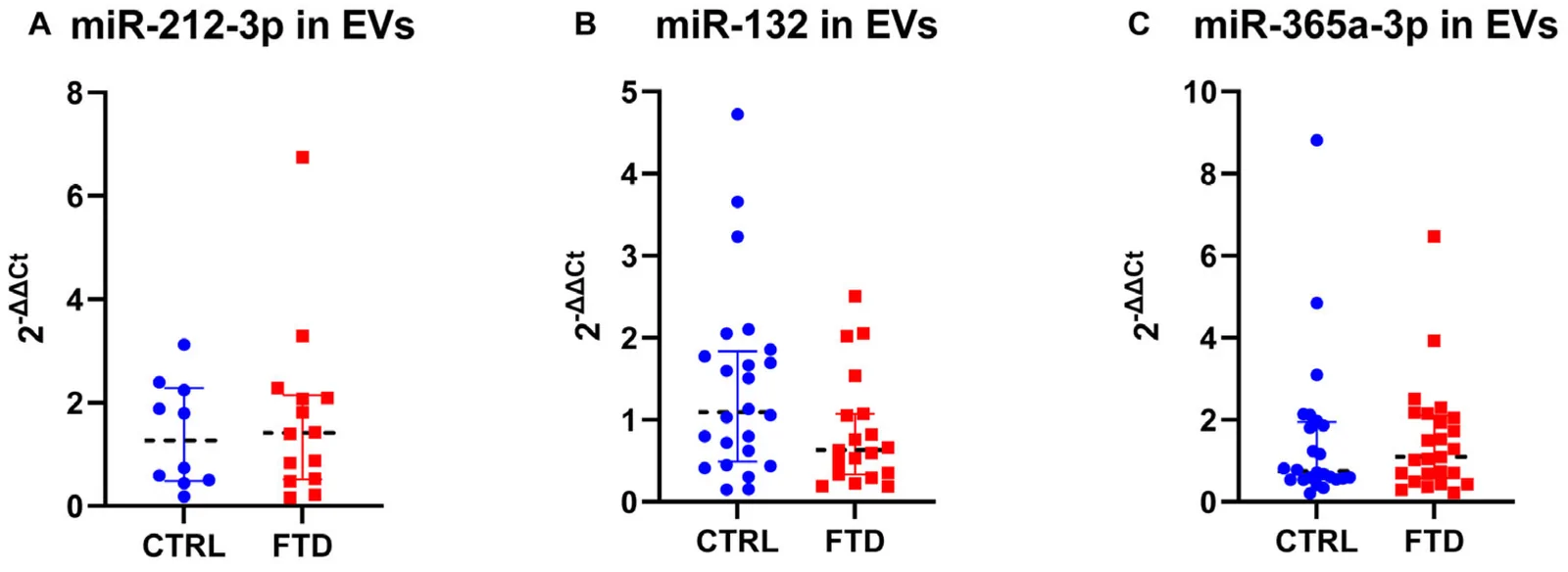
Validation through RT-qPCR of (**A**) miR-212-3p, (**B**) miR-132 and (**C**) miR-365a-3p in EVs of CTRL (in blue; n = 10, n = 24 and n = 24 respectively) and FTD patients (in red; n = 14, n = 19 and n = 25 respectively) using U6 snRNA as housekeeping gene. X axis: condition; Y axis: fold-expression indicated as 2^−∆∆Ct^. Mann–Whitney test has been used for statistical analyses. Data is expressed as medians and interquartile ranges. Dashed lines represent median value of each group. CTRL: healthy control. FTD: frontotemporal dementia. EVs: extracellular vesicles.

To assess the expression levels of the selected miRNAs, measurements were performed on EVs isolated from a cohort comprising 26 healthy controls (CTRLs) and 26 FTD patients. Relative expression data were normalized using U6 snRNA (Figure 2) and miR-16-5p (Figure S1) as endogenous controls (housekeeping genes). Samples exhibiting undetectable or unstable expression profiles were excluded from subsequent statistical analyses. Specifically, the final cohort sizes analyzed for each miRNA were as follows: 14 FTD and five CTRL patients for miR-212-3p; 19 FTD and 24 CTRL patients for miR-132; and 25 FTD and 24 CTRL patients for miR-365a-3p.

In line with the preliminary transcriptomic findings, miR-212-3p (Figure 2A) exhibited an up-regulation in the FTD group, whereas miR-132 (Figure 2B) was down-regulated. Similarly, miR-365a-3p (Figure 2C) showed a slight down-regulation in FTD patients compared to controls. Finally, correlation analysis using Spearman’s test was conducted to evaluate the potential impact of patient age on miRNA expression profiles; however, no significant association was observed between these variables (Figure S2).

### 3.3. DE miRNA Validation in PBMCs

**Figure 3:**
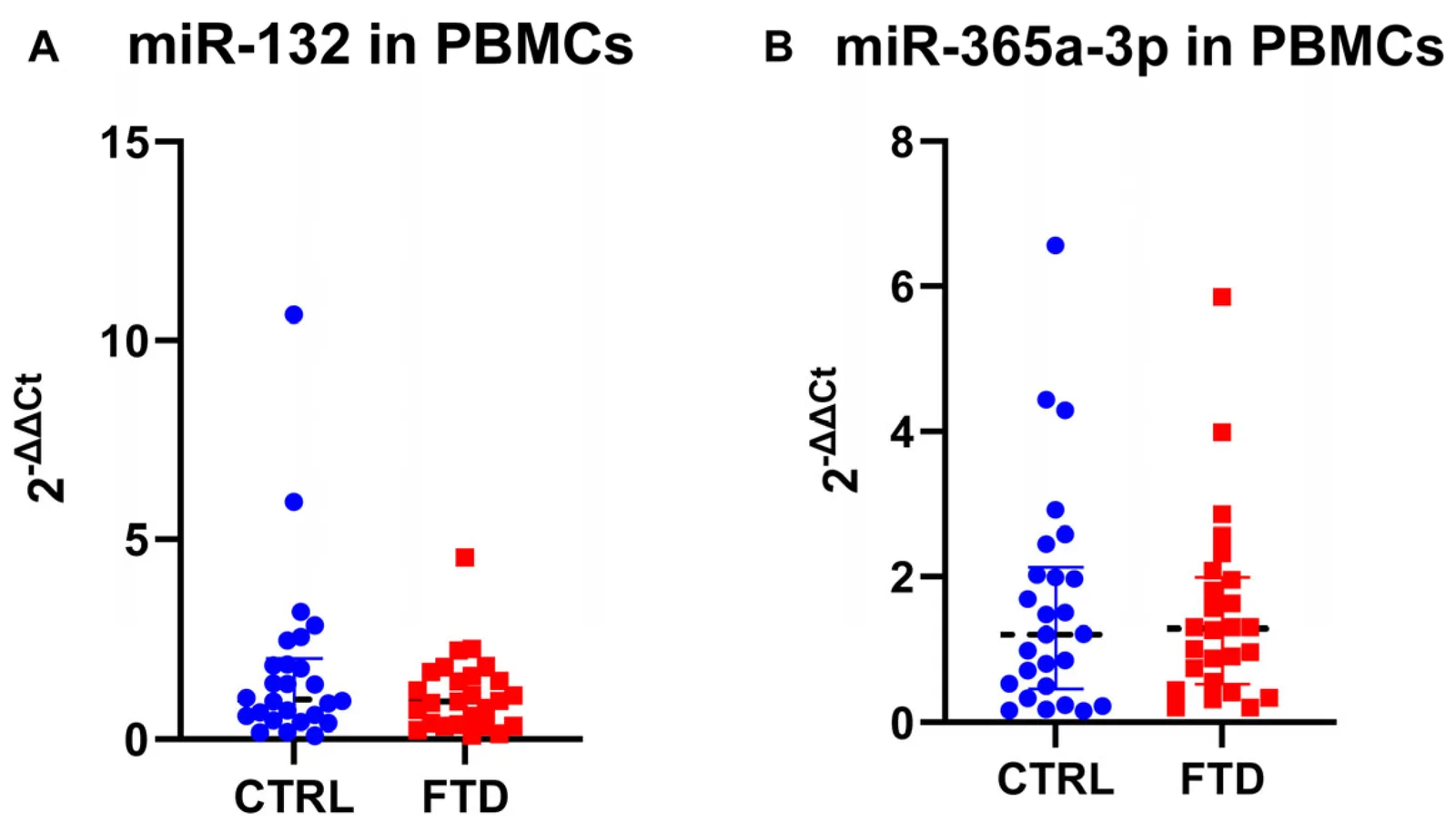
Validation through RT-qPCR of 2 deregulated miRNAs in PBMCs of CTRL (in blue; n = 26 for both miRNAs) and FTD patients (in red; n = 26 for both miRNAs) using U6 snRNA as housekeeping gene. (**A**) miR-132; (**B**) miR-365a-3p. X axis: condition; Y axis: fold-expression indicated as 2^−∆∆Ct^. Mann–Whitney test *has been used for statistical analyses.* Data is expressed as medians and interquartile ranges. *Dashed lines represent median value of each group.* CTRL: healthy control. FTD: frontotemporal dementia. PBMCs: peripheral blood mononuclear cells.

Based on their performance, miR-132 and miR-365a-3p were selected for further functional validation to assess their expression levels within PBMCs from a cohort of 26 CTRL and 26 FTD patients. MiR-212-3p was excluded because its expression levels determined by RT-qPCR did not correlate with the NGS data. Consistent with the previous phase, U6 snRNA (Figure 3) and miR-16-5p (Figure S3) were employed as endogenous reference controls for data normalization.

In agreement with NGS data, miR-132 (Figure 3A) was down-regulated in the FTD group. Similarly, miR-365a-3p (Figure 3B) exhibited a slight down-regulation in FTD patients compared to the control cohort. Correlations were assessed using Spearman’s rank correlation coefficient was performed to evaluate the relationship between patient age and miRNA expression profiles; however, no significant correlation was observed between these two variables (Figure S4).

### 3.4. Target Gene Selection

**Figure 4:**
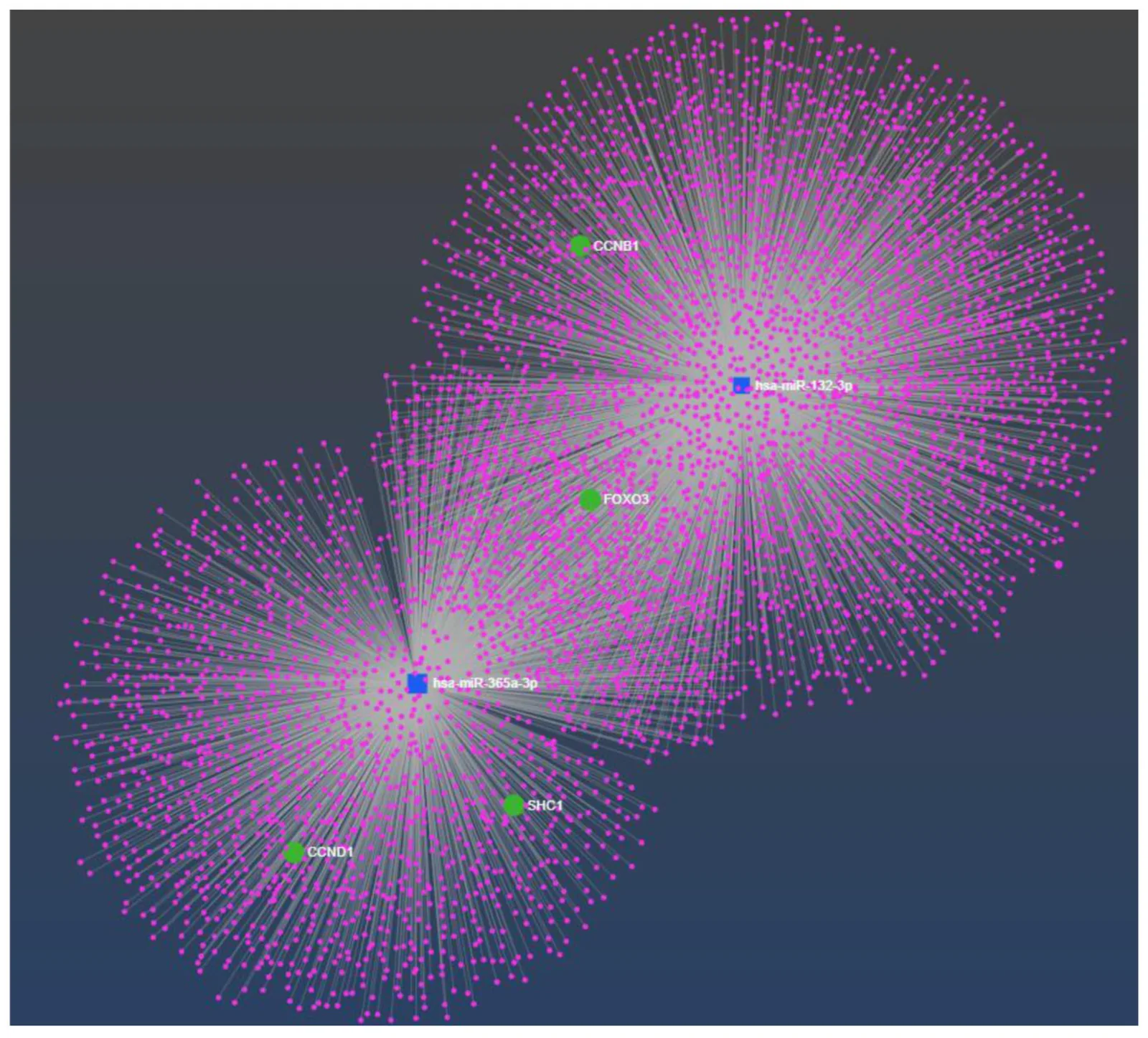
Network visualization of the target genes for the 2 miRNAs obtained using miRNet [25]. Nodes represent genes (pink: all targets; green: the selected target genes; blue: miRNAs).

Following the validation of the candidate miRNAs, their respective target genes were investigated to elucidate the functional roles these molecular regulators may play in the pathophysiology of FTD. Figure 4 provides a visual representation of the total target gene landscape for each miRNA. From this pool, specific target genes were selected based on a stringent multi-step workflow.

First, the interaction between miRNAs and their prospective targets had to be well-established and experimentally validated in the literature, which was verified using miRTarBase v9.0 [22]. Second, a literature review was conducted to confirm the involvement of these candidate genes in pathways characteristically associated with dementia. Based on these selection criteria, *CCNB1* and *FOXO3a* were selected as target genes for miR-132, while *CCND1* (or *CCND*) and *SHC1* were chosen as targets for miR-365a-3p.

### 3.5. Target Gene Validation in PBMCs

The expression levels of the selected target genes were evaluated in PBMCs from a cohort of 26 CTRL and 26 FTD patients. Beyond the previously described criteria, namely, robust literature validation via miRTarBase and involvement in dementia-related pathways, these targets were specifically expected to be up-regulated in PBMCs. This rationale stems from the classical mechanism of miRNAs, which act as negative regulators of mRNA expression; thus, a down-regulation of the regulatory miRNA should theoretically inversely correlate with an increase in its target mRNA levels. Following the exclusion of samples with undetectable or unstable amplification, the final analyzed cohorts were defined (e.g., 25 FTD and 26 CTRL samples for *CCND1*).

**Figure 5:**
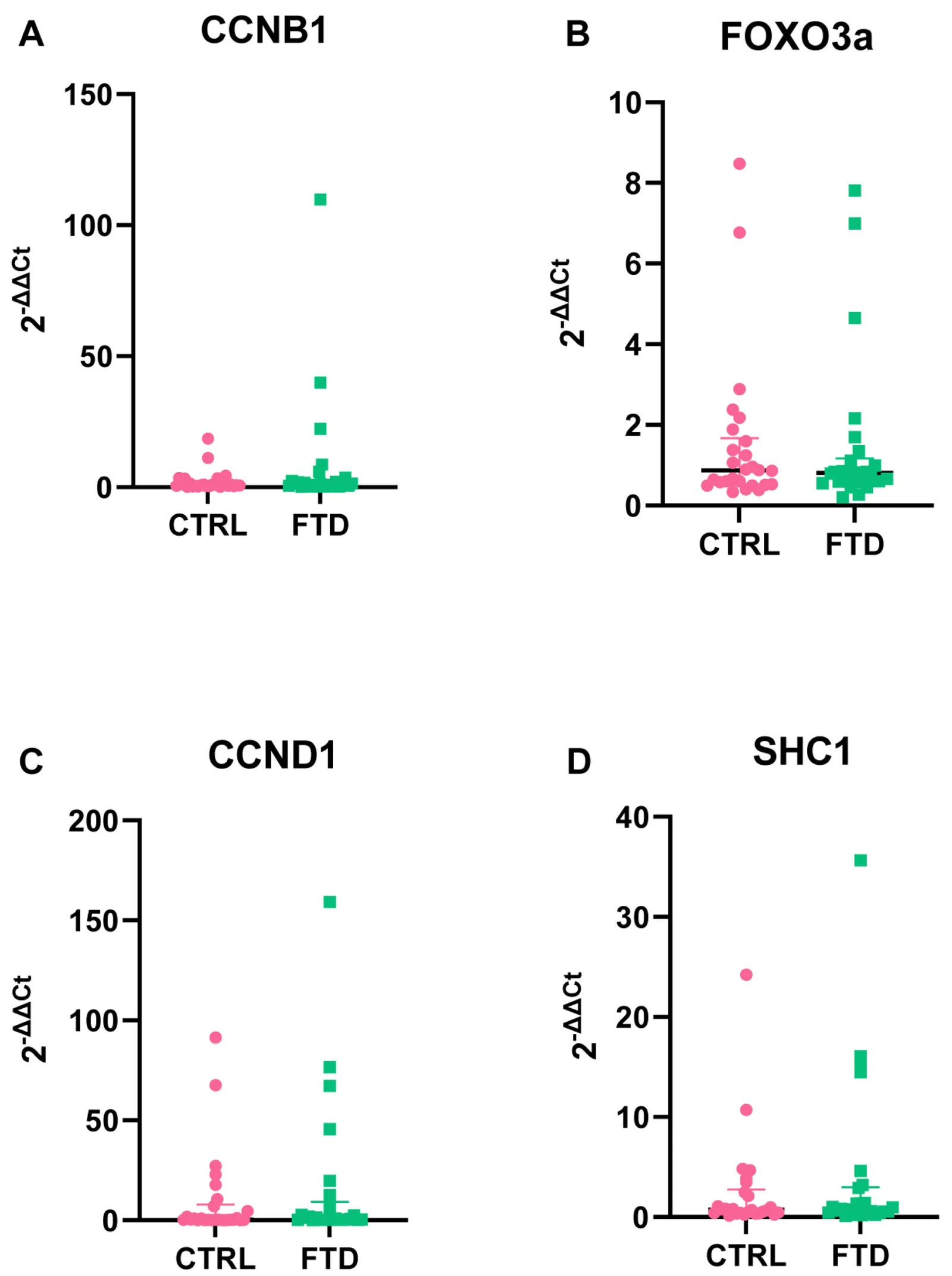
Validation through RT-qPCR of the selected target genes in PBMCs of CTRL (in pink; n = 26 for each gene) and FTD patients (in green; n = 26 for CCNB1, FOXO3a and SHC1, n = 25 for CCND1) using GAPDH as housekeeping gene. (**A**) CCNB1; (**B**) FOXO3a; (**C**) CCND1; (**D**) SHC1. X axis: condition; Y axis: fold-expression indicated as 2^−∆∆Ct^. Mann–Whitney test has been used for statistical analyses. Blacklines represent median value of each group. Data is expressed as medians and interquartile ranges. CTRL: healthy control. FTD: frontotemporal dementia.

The expression profiles of the dysregulated target genes in FTD are summarized in Table 3. For miR-132, *CCNB1* (Figure 5A) was found to be significantly up-regulated, whereas *FOXO3a* (Figure 5B) unexpectedly exhibited a down-regulation. Regarding miR-365a-3p, both selected targets, *CCND1* (Figure 5C) and *SHC1* (Figure 5D), were up-regulated in FTD patients. This robust up-regulation aligns with the preliminary RNA-seq data and literature evidence, further corroborating the findings from the two sequential validation phases in EVs and PBMCs. Finally, a Spearman’s correlation analysis revealed no significant correlation between patient age and the expression levels of any analyzed target gene (Figure S5).

The Table also reports the trend of predicted expression level of target genes based on miRNAs Log_2_FC and the observed expression level based on RT-qPCR. ↑: upregulated; ↓: downregulated.

## 4. Discussion

The research and discovery of new biomarkers is fundamental to better understanding and deepening our knowledge about every pathology, in this case FTD. In this context, investigation of EV cargo represents a promising approach to conduct this kind of study; in fact, EV-derived miRNAs can be considered as fluid-based biomarkers, making the diagnostic process easier and faster.

While in a previous work we performed EV characterization, NGS analysis and RT-qPCR validations [18] to find a EV-derived miRNA signature in different neurodegenerative diseases, the present study deliberately moves beyond descriptive high-throughput screening to focus specifically on FTD pathology. In this work, we selected FTD-specific miRNAs [18] in order to validate their expression levels and comprehensively investigate their relative target genes. This integrative approach allows us to dissect the precise functional roles that these differentially expressed small RNAs might have in the pathophysiology of the disease through the specific downstream gene networks they regulate.

An important limitation of studying miRNAs in FTD is the lack of information about their role in the pathology; in fact, there are many works that study miRNAs’ role in other neurodegenerative diseases such as AD, but it still has to be unraveled in FTD.

In this study, three deregulated miRNAs (miR-212-3p, miR-132 and miR-365a-3p) were analyzed in EVs and PBMCs. MiR-132 and miR-365a-3p showed better results and were prioritized for downstream target gene analysis. Following these validation steps, a total of two target genes for each miRNA were selected and validated to explore their potential involvement in FTD-related molecular pathways.

### 4.1. The miR-212-3p and miR-132 Axis: Disruption of Survival Signaling and Cell Cycle Control

MiR-212-3p regulates dendritic development [26] and inhibits apoptosis [27]. In this work, it appeared to be up-regulated, suggesting a pathological role in neurodegeneration that can be applied also to the FTD context; however, there is currently no research in the literature about its potential role in FTD. MiR-212 is often studied in conjunction with miR-132 as both are involved in neuronal survival pathways mediated by PTEN/AKT/FOXO3a signaling [28]. Nevertheless, this specific miRNA did not show consistent results in our experiments, and, for this reason, it was excluded from the subsequent target gene analyses.

In this work, miR-132 was found to be down-regulated, consistently with both NGS data and literature research about FTD [28,29]. Its roles in PI3K-Akt signaling [28], which is involved in the regulation of the cell cycle progression [30], and the consequent role in the FoxO signaling pathway since FOXO3a is a downstream target of the PI3K/Akt pathway [31] are known. The role of this miRNA has not been studied yet in FTD, but it has been seen that it might contribute to the deregulation of the Tau network, inflammation and synaptic function ending up with enhancing apoptosis [32,33,34]. These pathways are also central to FTD pathology, suggesting that miR-132 down-regulation may have broader implications across neurodegenerative conditions.

The miR-132 target genes investigated in this work are *CCNB1* and *FOXO3a*:

*CCNB1* appeared to be up-regulated, in agreement with transcriptomic data. While no study has ever correlated this gene with FTD, increased *CCNB1* expression has been reported in AD, MCI and ALS, suggesting a possible role in neurodegeneration [35,36,37]. This cyclin is involved with the transition from G2 phase to mitosis, meaning that it is responsible for the progression of the cell cycle and apoptosis [38]. Aberrant reactivation of the cell cycle in post-mitotic neurons has been described as a pathogenic mechanism in neurodegeneration, ultimately leading to mitotic catastrophe and neuronal death [39].

Similarly, *FOXO3a*, a transcription factor that stimulates apoptosis [28], was investigated as a direct miR-132 target. During the apoptotic process and in response to ROS, it can trigger the expression of typical apoptosis proteins like Bcl-XL, FasL and Bim [31]. Although its role in FTD remains unexplored, studies in AD indicate that FOXO3a increases the deposition of β-amyloid plaques and tau phosphorylation, aggravating the progression of the neuropathology [40,41]. The concomitant deregulation of miR-132 and its pro-apoptotic targets may suggest a severe imbalance in survival signaling pathways.

### 4.2. The miR-365a-3p Axis: Mitotic Re-Entry and Oxidative Stress Modulators

MiR-365a-3p showed a highly consistent trend between EVs and PBMCs. This miRNA is associated with the regulation of mitosis and apoptosis [42], and some studies have reported its deregulation in AD [43,44,45], although its role has not previously been investigated in FTD. Coherently with the miRNA down-regulation, its selected target genes, *SHC1* and *CCND1*, were found to be up-regulated in PBMCs.

*CCND1* encodes for cyclin D1 and controls the transition from G1 to S phase of the cell cycle [46,47]. It was also reported as an up-regulated gene in studies about other degenerative disorders such as AD, MCI and ALS [35,48,49]. For some still unknown reasons, neurons re-enter the cell cycle and *CCND1* is aberrantly expressed, leading to neuronal death [50]. This molecular mechanism might explain why there is massive neuronal loss in neurodegenerative diseases [36].

*SHC1* has never been studied in FTD, but it seems to regulate apoptosis, oxidative stress and the consequent ROS production [51], suggesting it may play a critical role in the main pathological processes that occur in FTD. The simultaneous deregulation of miR-365a-3p and genes controlling cell cycle and oxidative pathways further supports the hypothesis of systemic molecular alterations associated with neuronal vulnerability.

Overall, the deregulated miRNAs and their relative target genes are involved in oxidative stress but also in cell cycle control and apoptosis, confirming the results of the pathway analysis conducted in this work. Taken together, this cross-talk may reflect systemic alterations associated with neurodegeneration. Whether these changes represent compensatory responses to neuronal injury or active drivers of disease progression remains to be elucidated.

By moving from a baseline screening to a characterized miRNA–target axis, this study successfully addresses a major gap in the literature. The integrated analysis of EV-derived miRNAs and their downstream targets thus provides valuable, novel insights into the molecular mechanisms potentially associated with FTD pathophysiology.

## 5. Conclusions

Taken together, our data suggest that hsa-miR-132 and hsa-miR-365a-3p may contribute to the development of neurodegenerative disorders, particularly FTD, highlighting their potential utility as circulating plasma biomarkers for clinical translation. The convergence of these molecular alterations onto critical pathways controlling cell cycle re-entry, apoptosis, and oxidative stress strongly supports the presence of a systemic, post-transcriptional imbalance tightly associated with neurodegeneration. By integrating EV-derived miRNA profiling with peripheral target gene validation, our findings establish a robust framework for exploring RNA-mediated regulatory networks in FTD.

This work represents a crucial starting point; future investigations, particularly targeted in vitro mechanistic models, are now required to further dissect and deepen our understanding of the precise roles these miRNAs play in FTD pathology.

## 6. Limitations

A limitation of this study is the relatively small cohort size. In addition the number of successfully validated samples was lower than the total number of recruited participants given the fact that miRNAs in these samples were not detectable using RT-qPCR. As a result, the findings derived from these validation analyses should be considered preliminary and interpreted with appropriate caution as the limited sample size may have reduced the statistical power. Further validation will be essential to establishing the robustness of these findings.

It must be acknowledged that although NanoDrop is frequently used to quantify EV-derived RNA [52], it has limited accuracy for the low RNA concentrations usually obtained from plasma EVs. However all samples were processed using the same protocol for EV isolation, RNA extraction, quantification and reverse transcription, thereby minimizing systematic bias across experimental groups. RT-qPCR data were normalized using two endogenous reference controls (miR-16 and U6), reducing the potential impact of small inaccuracies in RNA quantification on the final expression analysis.

Another important limitation that must be recognized is that hemolysis is a pre-analytical factor in circulating miRNA studies, and miR-16-5p, used here as endogenous control, may be influenced by erythrocyte contamination. Although no specific hemolysis assessment was performed in the present study, all plasma samples were collected, processed, and stored according to the same protocol to minimize pre-analytical variability.

Normalization is a critical aspect of miRNA RT-qPCR analysis, particularly due to the lack of universally accepted endogenous reference. To improve the robustness of our analysis, we normalized the data using two commonly employed endogenous reference controls (miR-16-5p and U6 snRNA) rather than relying on a single normalizer. However, some statistical comparisons differed depending on the reference used; as a consequence, the results must be interpreted with caution.

## Figures and Tables

**Table 1:** Description of the cohorts analyzed in this work.

		EVs Validation		PBMCs Validation		Target Validation	
		CTRL	FTD	CTRL	FTD	CTRL	FTD
Number of samples		26	26	26	26	26	26
Age (mean)		65.2	66.0	64.2	65.7	64.2	65.7
Standard deviation (SD)		7.5	6.4	8.6	7.0	8.6	7.0
SEX (%)	M	54	54	46	50	46	50
	F	46	46	54	50	54	50

CTRL: healthy control; FTD: frontotemporal dementia. F = female. M = male.

**Table 2:** List of validated miRNAs and their relative Log_2_FC.

miRNA Name	Log_2_FC	Assay ID	miRNA Type
hsa-miR-212-3p	−6.83861674	000515	candidate miRNA
hsa-miR-132	-	000457	candidate miRNA
hsa-miR-365a-3p	−6.036483023	001020	candidate miRNA
U6 snRNA	-	001973	endogenous control
hsa-miR-16-5p	-	000391	endogenous control

**Table 3:** List of the miRNAs and their relative target genes deregulated in FTD.

miRNA	Target Gene	Predicted Target Expression	Observed Target Expression
miR-132	*CCNB1*	↑	↑
	*FOXO3a*	↑	↓
miR-365a-3p	*CCND1*	↑	↑
	*SHC1*	↑	↑

## Data Availability

The processed dataset that supports the findings of this study is openly available in Zenodo at http://doi.org/10.5281/zenodo.20718203.

## Supplementary Materials

The following supporting information can be downloaded at https://www.mdpi.com/article/10.3390/genes17080945/s1. Table S1: Details on the cohort of this study. Table S2: Oligonucleotide primer sequences of the selected target genes. Table S3A: Results of the Mann-Whitney test for miRnas validation in plasma. Table S3B: Results of the Mann-Whitney test for miRnas validation in PBMCs. Table S3C: Results of the Mann-Whitney test for miRnas validation in PBMCs. Figure S1: Validation through RT-qPCR. Figure S2: Correlation analysis using Spearman’s rank correlation coefficient. Figure S3: Validation through RT-qPCR. Figure S4. Correlations analysis using Spearman’s rank correlation coefficient. Figure S5: Correlation analysis using Spearman’s rank correlation coefficient.
