# Supplementary material for: MicroRNA Signature in Plasma-Derived Extracellular Vesicles in Frontotemporal Dementia
Source: Genes (Basel). 2026 Aug 13;17(8):945. doi: 10.3390/genes17080945 (PMC13511839; doi:10.3390/genes17080945)
Supplement: Supplementary file 1 [file genes-17-00945-s001.zip › Table S3A_S3B_S3C.pdf]

Table S3A. Results of the Mann-Whitney test for miRNAs validation in plasma of CTRL vs FTD using both U6 snRNA and miR-16-5p.

Biofluid	Biomarkers	CTRL	FTD	Mann-Whitney test
Plasma EVs (U6 snRNA)	miR-212-3p	10	14	0.8408
	miR-132	24	19	0.1043
	miR-365a-3p	24	25	0.555
Plasma EVs (miR-16-5p)	miR-212-3p	11	14	0.2441
	miR-132	24	19	0.5692
	miR-365a-3p	24	23	0.0282 *

The table presents p-values for the comparisons. * $p < 0.05$. The table also reports the number of samples exhibiting detectable and stable expression profiles used for the statistical analysis.

Table S3B. Results of the Mann-Whitney test for miRNAs validation in PBMCs of CTRL vs FTD using both U6 sn RNA and miR-16-5p.

Biofluid	Biomarkers	CTRL	FTD	Mann-Whitney test
PBMCs (U6 snRNA)	miR-132	26	26	0.4082
	miR-365a-3p	26	26	0.9167
PBMCs (miR-16-5p)	miR-132	26	24	0.0106 *
	miR-365a-3p	26	26	0.7099

The table presents p-values for the comparisons. * $p < 0.05$. The table also reports the number of samples exhibiting detectable and stable expression profiles used for the statistical analysis.

Table S3C. Results of the Mann-Whitney test for target genes validation in PBMCs of CTRL vs FTD using *GAPDH*. The table also reports the number of samples exhibiting detectable and stable expression profiles used for the statistical analysis.

Biofluid	Biomarkers	CTRL	FTD	Mann-Whitney test
PBMCs	*CCNB1*	26	26	0.3706
	FOXO3a	26	26	0.7961
	CCND1	26	25	0.2834
	SHC1	26	26	0.683

The table presents p-values for the comparisons. The table also reports the number of samples exhibiting detectable and stable expression profiles used for the statistical analysis.
