# Supplementary material for: MicroRNA Signature in Plasma-Derived Extracellular Vesicles in Frontotemporal Dementia
Source: Genes (Basel). 2026 Aug 13;17(8):945. doi: 10.3390/genes17080945 (PMC13511839; doi:10.3390/genes17080945)
Supplement: Supplementary file 1 [file genes-17-00945-s001.zip › Figure S1.pdf]

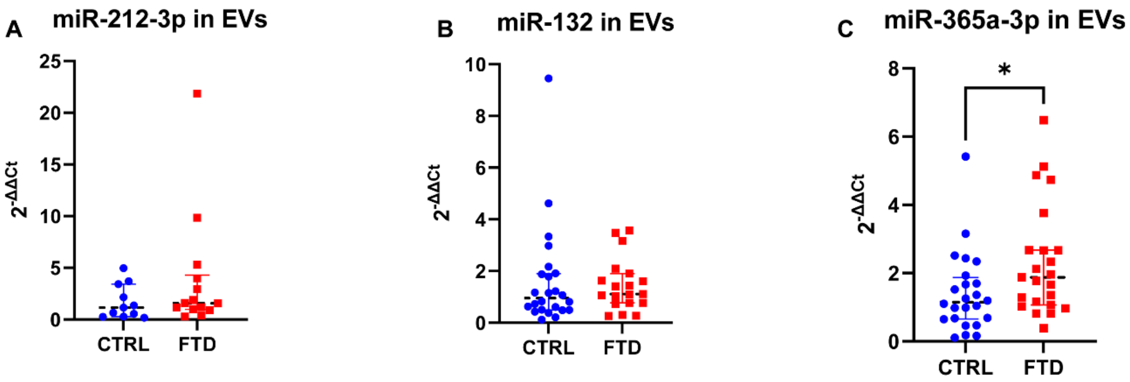

Figure S1. Validation through RT-qPCR of (**a**) miR-212-3p, (**b**) miR-132 and (**c**) miR-365a-3p in EVs of CTRL (in blue; n=11, n=24, n=24 respectively) and FTD patients (in red; n=14, n=19, n=25 respectively) using miR-16-5p as housekeeping gene. X axis: condition; Y axis: Fold-expression indicated as 2-ΔΔCt. Mann-Whitney test: * $p < 0.05$. Data is expressed as medians and interquartile ranges. CTRL: healthy controls. FTD: frontotemporal dementia. EVs: extracellular vesicles.
