# Supplementary material for: MicroRNA Signature in Plasma-Derived Extracellular Vesicles in Frontotemporal Dementia
Source: Genes (Basel). 2026 Aug 13;17(8):945. doi: 10.3390/genes17080945 (PMC13511839; doi:10.3390/genes17080945)
Supplement: Supplementary file 1 [file genes-17-00945-s001.zip › Figure S2.pdf]

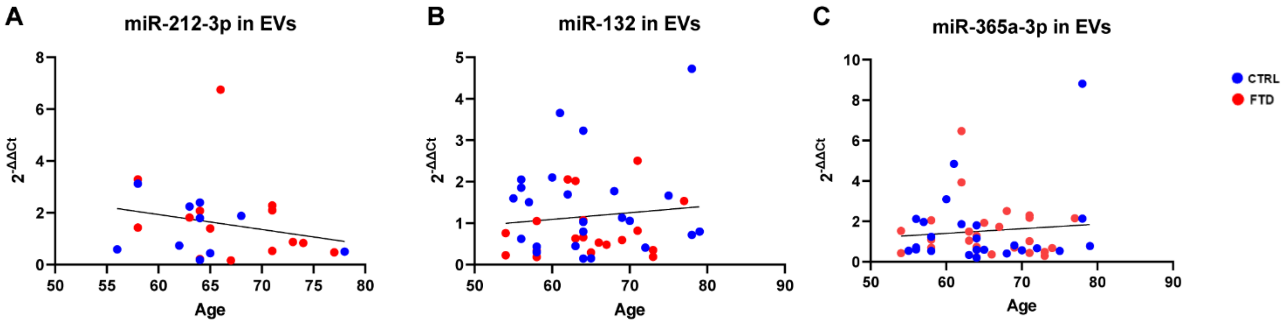

Figure S2. Correlations analysis using Spearman's rank correlation coefficient between age of CTRL (in blue) and FTD (in red) and (**a**) miR-212-3p (r=0-0,2652; p=0,2104), (**b**) miR-132 (r=0,0058; p=0,9704) and (**c**) miR-365a-3p (r=-0,05668, p=0,6989) expression level in EVs. The fitted line is shown for visualization only. X axis: Age; Y axis: Fold-expression indicated as 2-ΔΔCt. CTRL: healthy controls. FTD: frontotemporal dementia. EVs: extracellular vesicles.
