# Supplementary material for: MicroRNA Signature in Plasma-Derived Extracellular Vesicles in Frontotemporal Dementia
Source: Genes (Basel). 2026 Aug 13;17(8):945. doi: 10.3390/genes17080945 (PMC13511839; doi:10.3390/genes17080945)
Supplement: Supplementary file 1 [file genes-17-00945-s001.zip › Figure S3.pdf]

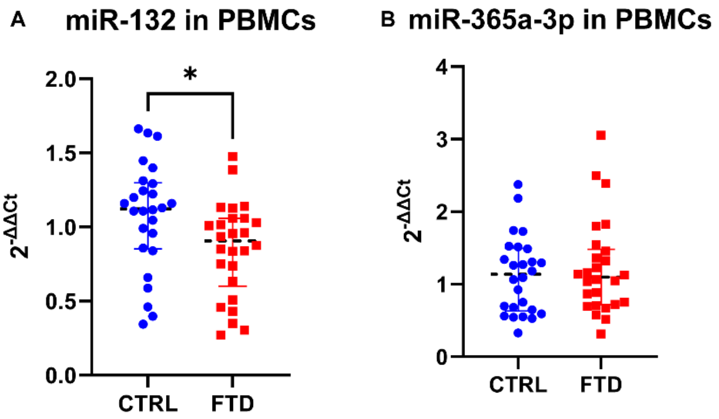


**Figure S3.** Validation through RT-qPCR of (**a**) miR-132 and (**b**) miR-365a-3p in PBMCs of CTRL (in blue; n=26 for both miRNAs) and FTD patients (in red; n=24, n=26 respectively) using miR-16-5p as housekeeping gene. X axis: condition; Y axis: Fold-expression indicated as 2-ΔΔCt. Mann-Whitney test: * $p < 0.05$. Data is expressed as medians and interquartile ranges. FTD: frontotemporal dementia. PBMCs: peripheral blood mononuclear cells.
