# Supplementary material for: MicroRNA Signature in Plasma-Derived Extracellular Vesicles in Frontotemporal Dementia
Source: Genes (Basel). 2026 Aug 13;17(8):945. doi: 10.3390/genes17080945 (PMC13511839; doi:10.3390/genes17080945)
Supplement: Supplementary file 1 [file genes-17-00945-s001.zip › Figure S4.pdf]

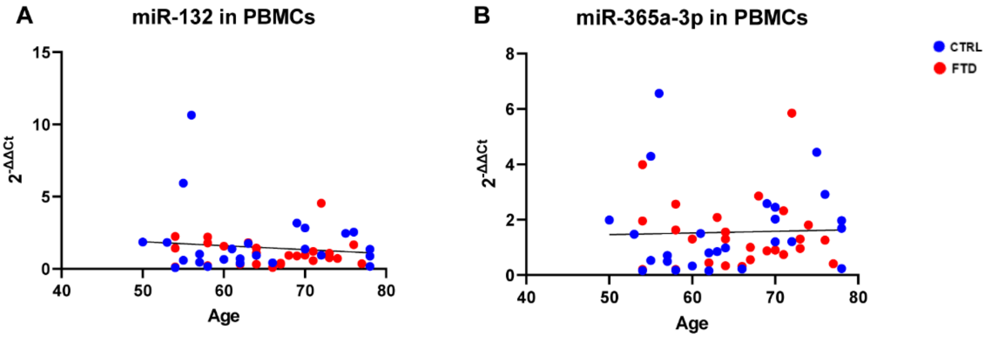


**Figure S4.** Correlations analysis using Spearman's rank correlation coefficient between age of CTRL (in blue) and FTD (in red) and (**a**) miR-132 (r=0,0003419; p=0,9981) and (**b**) miR-365a-3p (r=0,1416, p=0,3015) expression level in PBMCs. The fitted line is shown for visualization only. X axis: Age; Y axis: Fold-expression indicated as 2-ΔΔCt. CTRL: healthy controls. FTD: frontotemporal dementia. PBMCs: peripheral blood mononuclear cells.
