# Supplementary material for: MicroRNA Signature in Plasma-Derived Extracellular Vesicles in Frontotemporal Dementia
Source: Genes (Basel). 2026 Aug 13;17(8):945. doi: 10.3390/genes17080945 (PMC13511839; doi:10.3390/genes17080945)
Supplement: Supplementary file 1 [file genes-17-00945-s001.zip › Figure S5.pdf]

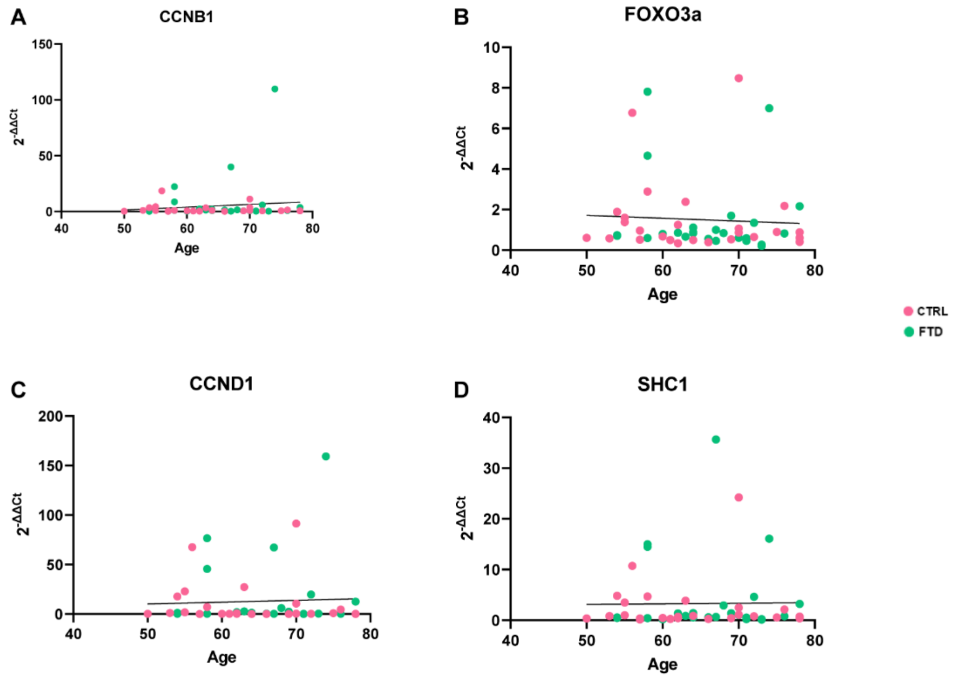

Figure S5. Correlations analysis using Spearman's rank correlation coefficient between age of CTRL (in pink) and FTD (in green) and (**a**) *CCNB1* (r=-0,001860; p<0,9896), (**b**) *FOXO3a* (r=-0,09708, p=0,4936), (**c**) *CCND1* (r=-0,01167; p<0,9352) and (**d**) *SHC1* (r=0,02633; p=0,8530) expression level in PBMCs. The fitted line is shown for visualization only. X axis: Age; Y axis: Fold-expression indicated as 2-ΔΔCt. CTRL: healthy controls. FTD: frontotemporal dementia.
