# Supplementary material for: MicroRNA Signature in Plasma-Derived Extracellular Vesicles in Frontotemporal Dementia
Source: Genes (Basel). 2026 Aug 13;17(8):945. doi: 10.3390/genes17080945 (PMC13511839; doi:10.3390/genes17080945)
Supplement: Supplementary file 1 [file genes-17-00945-s001.zip › Table S1.pdf]

**Table S1.** Details on the cohort of this study.

| Sample | Sex | Type | Disease's severity | Family history of dementia * | Genetic status * | Age | Age of onset | Disease duration | MRI * | FDG-PET * | MMSE | FAB | Medications* | Antidepressants* | Antipsychotics* | Systemic inflammatory, metabolic or malignant conditions* |
|---|---|---|---|---|---|---|---|---|---|---|---|---|---|---|---|---|
| Mon1 | F | MND | 1 | 0* | 0* | 64 | 61 | 3 | 1* | 1* | 25 | NA | 1* | 1* | 0* | 1* |
| Mon2 | M | bvFTD | 1 | 0* | 0* | 58 | 55 | 3 | 1* | 1* | 25 | 0 | 1* | 1* | 1* | 1* |
| Mon3 | M | nfvPPA | 1 | 1* | 0* | 69 | 68 | 1 | 1* | 1* | 18 | 4 | 0* | 0* | 0* | 1* |
| Mon4 | F | nfvPPA | 1 | 0* | 0* | 71 | 68 | 3 | 1* | 1* | 14 | NA | 1* | 0* | 0* | 1* |
| Mon5 | F | nfvPPA | 1 | 0* | 0* | 76 | 75 | 1 | 1* | 0* | 22 | 5 | 0* | 0* | 0* | 1* |
| Mon6 | F | bvFTD | 1 | 0* | 0* | 54 | 53 | 1 | 1* | 1* | 25 | 8 | 1* | 1* | 0* | 1* |
| Mon7 | M | bvFTD | 2 | 1* | 0* | 73 | 66 | 7 | 1* | 1* | 10 | 3 | 1* | 0* | 0* | 1* |
| Mon8 | M | bvFTD | 1 | 0* | 0* | 64 | 61 | 3 | 1* | 1* | 20 | 8 | 1* | 1* | 0* | 1* |
| Mon9 | F | MND | 1 | 1* | 0* | 63 | 62 | 1 | 1* | 0* | 27 | 9 | 1* | 1* | 0* | 1* |
| Mon10 | F | MND | 2 | 0* | 0* | 74 | 72 | 2 | 1* | 0* | 27 | NA | 1* | 1* | 1* | 1* |
| Mon11 | M | bvFTD | 1 | 1* | 0* | 71 | 71 | 0 | 1* | 1* | 30 | 14 | 1* | 0* | 1* | 1* |
| Mon12 | M | bvFTD | 1 | 1* | 0* | 58 | 57 | 1 | 1* | 0* | 30 | 15 | 1* | 0* | 0* | 1* |
| Mon13 | F | bvFTD | 2 | 0* | 0* | 54 | 52 | 2 | 1* | 1* | 19 | 11 | 1* | 1* | 1* | 1* |
| Mon14 | M | bvFTD | 1 | 0* | 0* | 77 | 74 | 4 | 1* | 1* | 21 | NA | 1* | 1* | 1* | 0* |
| Mon15 | M | bvFTD | 1 | 1* | 0* | 58 | 57 | 1 | 1* | 1* | 27 | NA | 1* | 1* | 0* | 0* |
| Mon16 | F | bvFTD | 2 | 1* | 0* | 62 | 57 | 5 | 1* | 0* | 15 | 4 | 1* | 0* | 0* | 1* |
| Mon17 | F | bvFTD | 2 | 0* | 0* | 66 | 63 | 3 | 1* | 0* | 11 | NA | 1* | 0* | 1* | 1* |
| Mon18 | M | bvFTD | 1 | 1* | 0* | 71 | 69 | 3 | 1* | 0* | 26 | NA | 1* | 0* | 1* | 1* |
| Mon19 | M | bvFTD | 1 | 0* | 0* | 73 | 71 | 3 | 1* | 1* | 28 | 12 | 1* | 1* | 1* | 0* |
| Mon20 | F | bvFTD | 2 | 1* | 0* | 68 | 66 | 1 | 1* | 0* | 15 | 5 | 1* | 1* | 0* | 1* |

| Sample | Sex | Type | Disease's severity | Family history of | Genetic status * | Age | Age of onset | Disease duration | MRI * | FDG-PET * | MMSE | FAB | Medications * | Antidepressants* | Antipsychotics* | Systemic inflammatory, metabolic or malignant |
|---|---|---|---|---|---|---|---|---|---|---|---|---|---|---|---|---|
| Mon21 | F | bvFTD | NA | NA | 0* | 63 | NA | NA | 1* | NA | NA | NA | NA | NA | NA | NA |
| Mon22 | F | bvFTD | 1 | 0* | 0* | 62 | 60 | 2 | 1* | 0* | 24 | 13 | 1* | 0* | 1* | 1* |
| Mon23 | M | bvFTD | 2 | 1* | 0* | 67 | 59 | 8 | 1* | 0* | 20 | 7 | 1* | 1* | 1* | 1* |
| Mon24 | M | bvFTD | 2 | 0* | 0* | 64 | 60 | 4 | 1* | 0* | NA | NA | 1* | 0* | 1* | 1* |
| Mon25 | M | bvFTD | 2 | 0* | 0* | 65 | 62 | 3 | 1* | 1* | 16 | 7 | 1* | 0* | 1* | 1* |
| Mon26 | M | lvPPA | 2 | 0* | 0* | 71 | 66 | 5 | 1* | 1* | NA | NA | 1* | 0* | 1* | 1* |
| Mon27 | F | bvFTD | 1 | 1* | 0* | 60 | 60 | 0 | 1* | 0* | 28 | 12 | 1* | 1* | 1* | 1* |
| Mon28 | F | bvFTD | 2 | 1* | 0* | 54 | 48 | 6 | 1* | 1* | 11 | 9 | 1* | 1* | 1* | 1* |
| Mon29 | M | MND | 1 | 0* | 0* | 72 | 69 | 4 | 1* | 1* | 29 | 15 | 1* | 1* | 0* | 1* |
| Mon30 | F | bvFTD | 1 | 1* | Granulin | 67 | 67 | 0 | 1* | 0* | 27 | 10 | 1* | 0* | 1* | 1* |
| Mon31 | M | bvFTD | 1 | 0* | 0* | 70 | 67 | 3 | 1* | 1* | 26 | NA | 1* | 0* | 1* | 1* |

NA: not available. * Legend= 0: no, 1:yes; Disease’s severity: 1: mild, 2:severe
