# Supplementary material for: MicroRNA Signature in Plasma-Derived Extracellular Vesicles in Frontotemporal Dementia
Source: Genes (Basel). 2026 Aug 13;17(8):945. doi: 10.3390/genes17080945 (PMC13511839; doi:10.3390/genes17080945)
Supplement: Supplementary file 1 [file genes-17-00945-s001.zip › Table S2.pdf]

Table S2. Oligonucleotide primer sequences of the selected target genes.

Target name	Forward	Reverse	Target type
CCNB1	ACAAGCCTGTCCCCATCAAG	CACTGGCGTTCACAAGGTTG	candidate target
FOXO3a	GCCATTCTCATCGGGTCCTC	TGATGGCCCCCTCTGTGTTA	candidate target
CCND1	AAATACCTGGAGTGCTCGGC	TCGGATCGCTTCGTCAAACA	candidate target
SHC1	ATCCTGGAATGTTGGCTGCA	CCTGGGTTTGGGTACTGCAT	candidate target
GAPDH	AGGAGTAAGACCCCTGGACC	GGGGAGATTCAGTGTGGTGG	endogenous control
